# *Dictyostelium purpureum* var. *pseudosessile,* a new variant of dictyostelid from tropical China

**DOI:** 10.1186/s12862-019-1407-2

**Published:** 2019-03-14

**Authors:** Pu Liu, Yue Zou, Jiangan Hou, Steven L. Stephenson, Yu Li

**Affiliations:** 1 Engineering Research Center of Chinese Ministry of Education for Edible and Medicinal Fungi, Jilin Agricultural University, Changchun, 130118 People’s Republic of China; 2 Department of Biological Sciences, University of Arkansas, Fayetteville, AR 72701 USA

**Keywords:** Dictyostelids, Taxonomy, Ontogeny, Phylogeny

## Abstract

**Background:**

Dictyostelid cellular slime molds (dictyostelids) are microscopic throughout their entire life cycle. The vegetative phase consists of single-celled amoeboid forms which live in the soil/leaf litter microhabitat of fields and forests along with animal dung, where they feed upon bacteria and other microbes, grow, and multiply until the available food supply is exhausted. When this happens, the amoeboid forms aggregate together in large numbers to form multi-celled pseudoplasmodia, which then give rise to fruiting bodies (sorocarps) that consist of supportive stalks and unwalled sori containing propagative spores.

**Results:**

*Dictyostelium purpureum* var. *pseudosessile*, a new variant of dictyostelid, is described herein, based on morphological features and molecular data. This new variant was isolated from soil samples collected in two tropical areas of China. The complete spore-to-spore life cycle of this species, which required 50 h, including spore germination, myxamoebae, cell aggregation, pseudoplasmodium, and sorocarp formation, was documented. Descriptions and illustrations are provided for this species based on our collections. Data from ontogeny, morphology and phylogeny analyses (SSU) of *D. purpureum* var. *pseudosessile* confirm that it is a Group 4 species according to the newly proposed classification of dictyostelids.

**Conclusions:**

Our results suggest that the violet sori, widens at the midpoint of sorophore and simple recurved sorophore bases represent the prominent features for the new variant *D. purpureum* var. *pseudosessile*. The latter is a Group 4 species now known from two tropical areas of China where dictyostelids remains understudied.

**Electronic supplementary material:**

The online version of this article (10.1186/s12862-019-1407-2) contains supplementary material, which is available to authorized users.

## Background

Dictyostelid cellular slime molds are key inhabitants of the soil and leaf litter layer of fields and forests, along with animal dung, where they feed mostly on bacteria [1–3]. Protists such as dictyostelids are important agents in bringing about quantitative changes to the bacterial microflora of soils and apparently play a role in maintaining the balance that exists between bacteria and other microbes in the soil [4, 5]. Dictyostelids are members of Amoebozoa, a branch of eukaryotes separate from plants, fungi and animals. Their cells lack cell walls and resemble animal cells in organization, except for the presence of a contractile vacuole [6].

Since the first dictyostelid was described by Brefeld [7], more than 100 species have been reported and classified into one class, one order, two families and four genera [8]. These species are distinguished morphologically largely on the basis of differences in sorophore composition and branching pattern. However, a phylogenetic analysis based on 18S ribosomal RNA (18S rRNA) and α-tubulin indicated that the traditional morphological based classification traditionally used for these organisms did not hold up. The genera that had always been used for dictyostelids were not found to be monophyletic, and the various species were separated first into four groups [9] and then into eight groups [10], respectively. Furthermore, a new classification, based on unique 18S rRNA sequence signatures, was proposed, and this provided an additional new insight into the taxonomy of the dictyostelids. As a result of this new classification, two families, nine genera and 92 new combinations were recognized at the level of species and variety [11].

The Hainan Province and Xishuangbanna region of Yunnan Province are both located south of Tropic of Cancer and are characterized by a tropical monsoon climate. However, studies of dictyostelids in this region are exceedingly limited and consist of only a single previous report of two species of dictyostelids from the Hainan Province [12]. To fill in this gap, focus of the present study was directed towards the morphology, ontogeny, and phylogeny of a new variant of dictyostelids recovered from soil samples collected at these two sites.

## Results

**Fig. 1:**
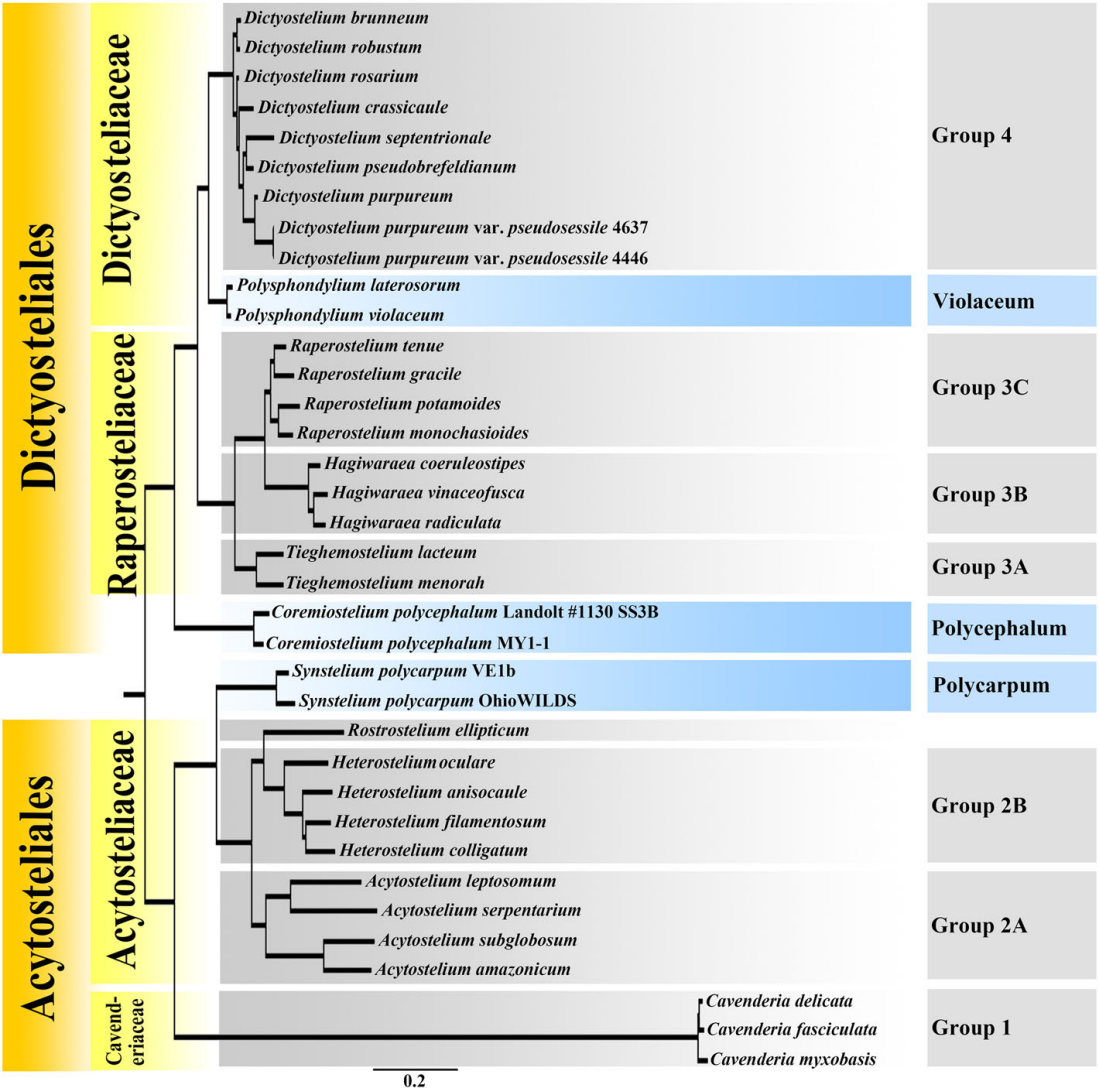
Phylogenetic tree of dictyostelids based on SSU rRNA and portions of the SSU rRNA gene alignment, showing the molecular signatures of *Dictyostelium purpureum* var. *pseudosessile*

After being processed, two isolates representing one new tropical variant of dictyostelids were yielded, with one isolate obtained from the Xishuangbanna Tropical Botanical Garden and a second isolate from the Qixianling Hot Springs National Forestry Park. It required 50 h for this species to complete its life cycle. Phylogenetic studies of the nuclear SSU rDNA showed that the new variant is a member of Group 4 (Fig. 1), based on the concepts of Schaap et al. [9], Romeralo et al. [10], and Sheikh et al. [11].

### Description of the new variant

**Fig. 2:**
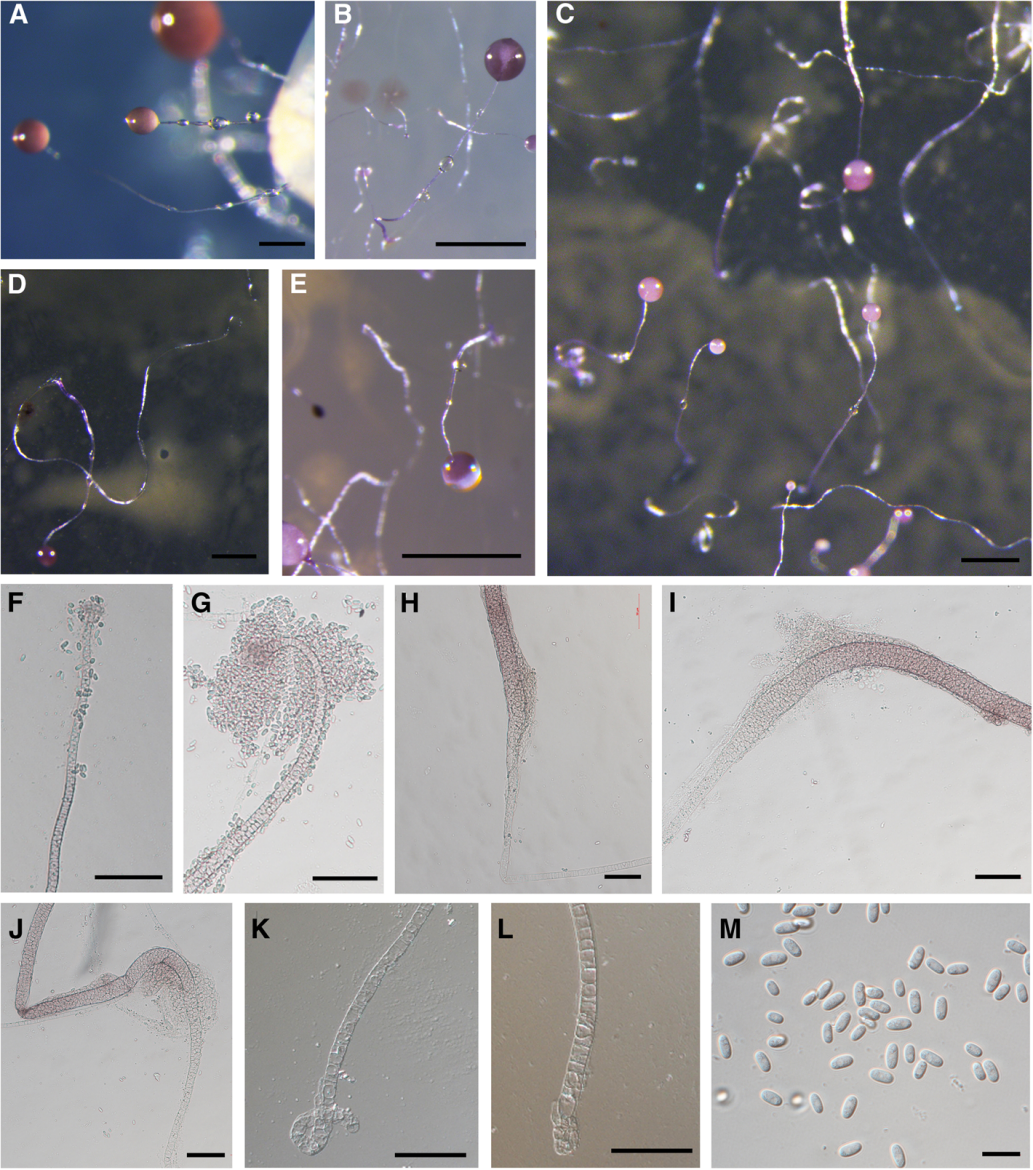
Morphological features of *Dictyostelium purpureum* var. *pseudosessile.* **a**–**e** Sorocarps. **f**,**g** Sorophore tip. **h**–**j** Mid-point of the sorophore. **k**,**l** Sorophore base. **m** Spores. Scale bars: **a**: 200 μm, **b**,**e**: 1 mm, **c**: 0.5 mm, **d**: 0.5 mm; **f**–**l**: 20 μm, **m**: 10 μm

***Dictyostelium purpureum*** **var.** ***pseudosessile*** Li Y., P. Liu, Y. Zou, S.L. Stephenson et J. Hou, sp. nov. (Fig. 2).

MycoBank: MB825215.

#### Holotype

China. Hainan Province, Qixianling Hot Springs National Forestry Park (109°41′E and 18°42′N), located at an elevation of approximately 350 m, from a soil sample collected within a broadleaf rainforest, 13 January 2015, HMJAU MR279 (4446).

#### Diagnosis

Sorocarps with several sessile droplets of water on one sorophore, gregarious, erect or semi-erect, unbranched, 4.3–7.3 mm high. Sorophore sinuous, white or violet, prostrate on the agar, widens at the midpoint of the whole sorophore, bases recurve, tips capitate. Sori violet, globose. Spores oblong to elliptical, without obvious polar granules, 5.0–10 × 2.5–5 μm. Aggregation streamed.

#### Etymology

From the Latin *pseudosessile*, referring to the several sessile droplets of water occurring on one sorophore.

#### Description

When cultured at 23 C on non-nutrient agar with *E. coli*, **sorocarps** (Fig. 2–a–e) generally gregarious, erect or semi-erect, unbranched, commonly 4.3–7.3 mm high (average 5.7 mm) with a variable number of sessile droplets of water occuring on one sorophore. **Sorophore** sinuous, white or violet, nearly half of the lower portion of the sorophore prostrate on the surface of the agar, the other upper portion of the sorophore erect or semi-erect on the agar. The erect or semi-erect portion of the sorophore widens (Fig. 2–h–j) at the base and general consists of several tiers of cells, but the base (Fig. 2–k, l) of the prostrate portion of the sorophore recurves and usually consists of one or two tiers of cells (10–37.5 μm diam, average 18.5 μm). Tips (Fig. 2–f, g) capitate and consisting of one or two tiers of cells (5–10 μm diam, average 7.5 μm). **Sori** violet, globose to lemon-type, commonly 135–325 μm diam (average 236 μm). **Spores** (Fig. 2–m) oblong to elliptical, 5.0–10 × 2.5–5 μm, without obvious polar granules. **Aggregations** (0.7–4 mm) generally with asymmetrical streams. Early sorogens fusiform to coniform, migrate with stalk formation.

#### Other specimens examined

China. Yunnan Province, Chinese Academy of Sciences, Xishuangbanna Tropical Botanical Garden (101°25′ E and 21°41′ N), located at an elevation of approximately 570 m, from a soil sample collected from beneath *Hydnocarpus annamensis* (Gagnep.) M. Lescot et Sleum, 20 January 2015, HMJAU MR273 (4637).

### Life cycle of *D. purpureum* var. *pseudosessile*

**Fig. 3:**
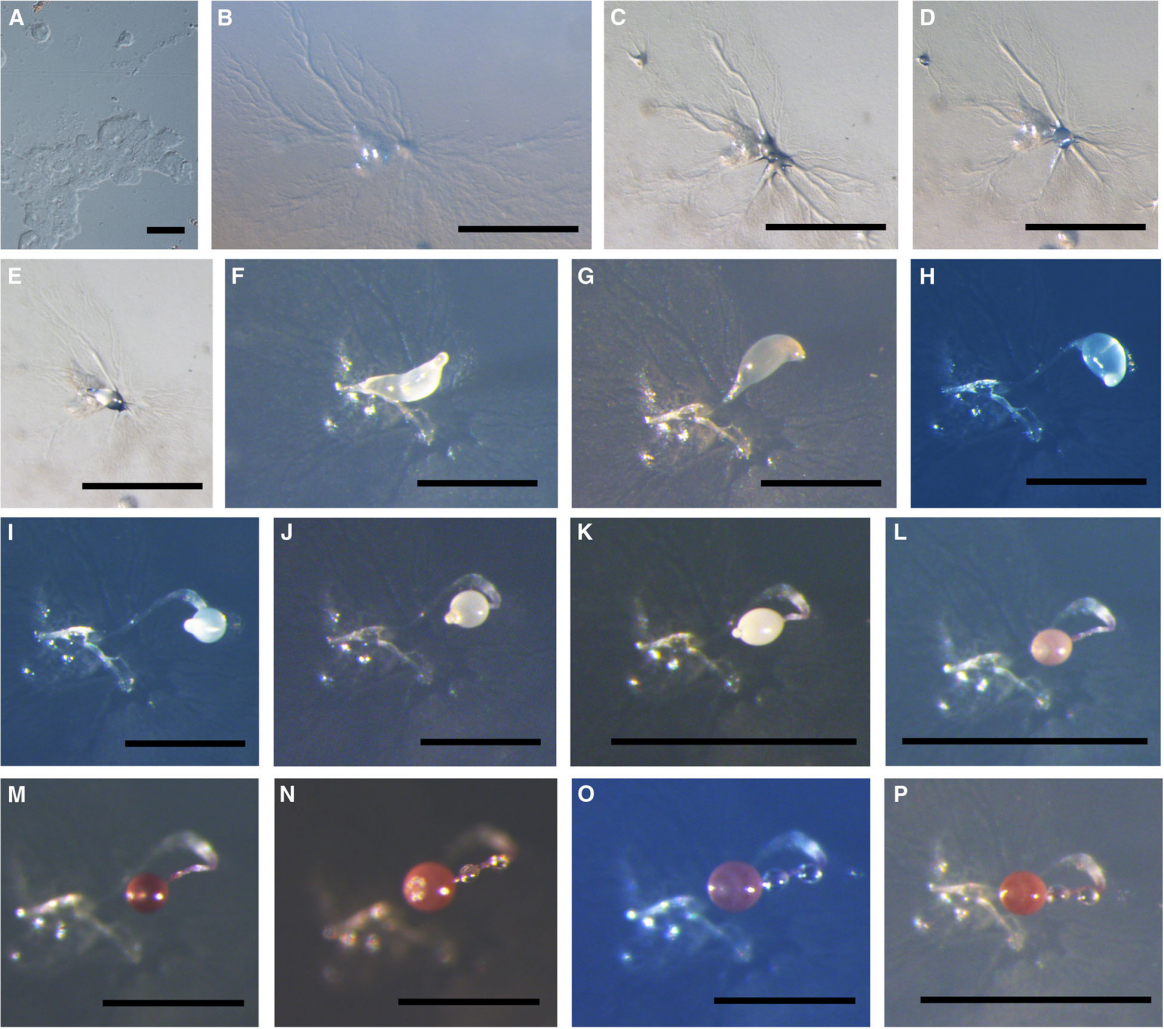
Life cycle of *Dictyostelium purpureum* var. *pseudosessile*. **a** Myxamoebae. **b**–**d** Aggregations. **e**–**i** Formation of pseudoplasmodia. **j**–**k** Young sorocarp. **l**–**p** Fruiting of sorocarp. Scale bars: **a**: 20 μm, **b**–**e**,**k**,**l**,**p**: 1 mm, **f**–**j**,**m**–**o**: 500 μm

Spores become larger when germination occurs; the latter begins with the appearance of a minute pore dissolved in the spore wall, and several myxamoebae are released from different spores in concert (Fig. 3–a). Myxamoebae are colorless, transparent, and irregular. A mass of myxamoebae aggregates to form a young aggregation center 32.5 h after the spores have been inoculated on agar (Fig. 3–b). Two hours later, the central portion of the aggregation begins to form a small central point with a prominent stream of cells (Fig. 3–c). After the aggregating of more and more myxamoebae, all myxamoebae aggregate at one central point, the prominent central point rises up and with fewer streams 35.5 h after the spores have been inoculated on agar (Fig. 3–d). One and a half hours later, the central cell aggregations rise up and begin to form pseudoplasmodia with indistinct sorophores (Fig. 3–e). Two and a half hours later, the sorophores begin to form (Fig. 3–f). Sorophores grow longer (Fig. 3–g, h), which gives them the appearance of a fruiting sorophore (Fig. 3–i) at 42 h, pseudoplasmodia form and undergo a very short period of movement. The sorophore is slightly curved, half of which is prostrate on the surface of the agar. After the formation of the sorophore, the sori begin to grow gradually (Fig. 3–j, k) to form globose sori at 47 h (Fig. 3–l), the color changes from white (Fig. 3–j), pale yellow (Fig. 3–k), pale violet (Fig. 3–l), to violet (Fig. 3–m). Forty-eight h after inoculating the spores on the agar, colorless sessile droplets of water begin to form on the sorophore, these are oblong (Fig. 3–n) at first but gradually change from oblong to globose (Fig. 3–o), and the sorocarps finally fruit at 50 h (Fig. 3–p). Sorocarps are solitary, erect or semi-erect and lack of branches.

## Discussion

**Fig. 4:**
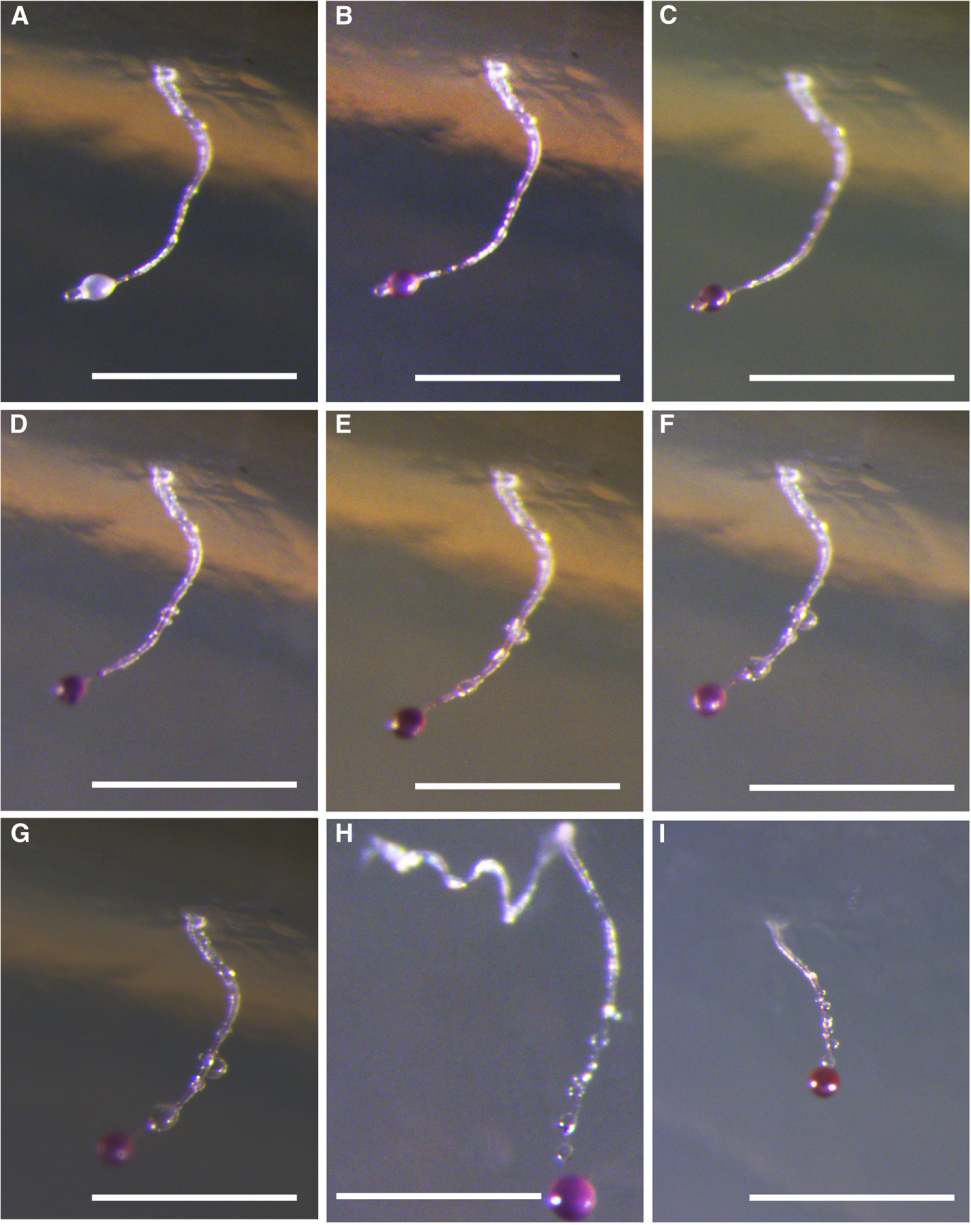
*Dictyostelium purpureum* var. *pseudosessile*. **a**–**g** Fruiting process of sorocarps, each from them were photographed in one hour. **h, i** Other sorocarps. Scale bars: **a**–**g**: 500 μm, **h, i**: 1 mm

Morphologically, *Dictyostelium purpureum* var. *pseudosessile* is a variant of *D. purpureum* Olive characterized particularly by its colorless sessile droplets of water (Fig. 4) borne at intervals along the upright sorophores and simple recurved sorophore bases. Although *D. purpureum* var. *pseudosessile* forms a clade with *D. purpureum* (Fig. 1), *D. purpureum* [5, 11] has sorophores with basal disks, whereas the new variant somewhat widens at the midpoint of sorophore but no basal disks. In order to discriminate *D. purpureum* var. *pseudosessile* with *D. purpureum* deeply, we mixed these strains on one agar plate according to the method of Sathe et al. [13], we found the aggregations and pesudoplasmodia have different tips, then finally form both sorocarps of *D. purpureum* var. *pseudosessile* and *D. purpureum* on one plate (Additional file 1: Fig. S1). The SSU sequences of *D. purpureum* var. *pseudosessile* and *D. purpureum* have 97% identity (Additional file 1: Fig. S2). From the observations of the life cycle of *D. purpureum*, no sessile droplets of water on the sorophore were found during this process (Additional file 1: Fig. S3). Therefore, *D. purpureum* var. *pseudosessile* is different with *D. purpureum* in both morphology and phylogeny.

*Dictyostelium purpureum* var. *pseudosessile* is rare but is known to occur in two regions of tropical China. The morphological and molecular features of *D. purpureum* var. *pseudosessile* are considerably different compared to *D. purpureum* mentioned above, with the most prominent features of the new variant being the prostrate sorophore, widens at the midpoint of sorophore and simple recurved sorophore bases. The entire life cycle of *D. purpureum* var. *pseudosessile* extends over 3 d (50 h). The spores germinated and released myxamoebae, and aggregations formed 32.5 h after inoculation. Pseudoplasmodium formation required 42 h. Following this, the sorophores and sori begin to grow in an orderly manner and finally formed fruiting sorocarps after 50 h.

The most important contribution of this paper is that it extends our knowledge of the distribution and ecology of dictyostelids in a region of the world where these organisms remain understudied. It seems likely that additional sampling in tropical areas of China will yield other species new to science or already described species which are not yet known to occur in China.

## Conclusions

Reported herein are two isolates, each from a different area of tropical China, which represented a new variant of *Dictyostelium purpureum*. Our discovery of this new variant (*D. purpureum* var. *pseudosessile*) from tropical areas of China expands the biogeographical range of dictyostelids. Molecular phylogenetic analyses of the two isolates revealed that they do not belong to any previously described species. We propose that the two new isolates belong to the same species, which is characterized by violet sori, widens at the midpoint of sorophore and simple recurved sorophore bases. This new variant required 50 h to complete its entire life cycle, incuding spore germination, aggregation formation, pseudoplasmodium formation, and sorocarp formation.

## Methods

### Study site ecology

Soil samples used for the isolation of dictyostelids were collected in two tropical areas of China in January 2015. These were the Xishuangbanna Tropical Botanical Garden of the Chinese Academy of Sciences located in Yunnan Province and Qixianling Hot Springs National Forestry Park located in Hainan Province. The Xishuangbanna Tropical Botanical Garden is situated in the southwestern portion of China, whereas the Qixianling Hot Springs National Forestry Park occurs in the southernmost portion of China. Both of these two localities are located south of the Tropic of Cancer and are characterized by a tropical monsoon climate, with an annual temperatures of 21.4 C and 23.0 C, respectively.

### Sampling

Samples, each consisting of approximately 30–50 g of material, were collected and placed in sterile whirl-pack plastic bags. In most instances, at least five samples were collected from each vegetation type at each locality. Afterwards, these samples were returned to the laboratory as soon as possible, following the recommendations of Cavender and Raper [3]. Each sample bag was numbered and the sample itself preserved at 4 C in the herbarium of the Mycological Institute of Jilin Agricultural University (HMJAU), Changchun, China.

### Isolation and cultivation

The isolation methods used followed those described by Cavender and Raper [3]. Each sample was weighed and diluted for an initial dilution of 1:10 by adding ddH_2_O. This solution was shaken to disperse the material and to suspend the amoebae and spores of dictyostelids present. Afterwards, a 0.5 mL aliquot of the solution was added to each of five duplicate culture plates prepared with hay infusion agar [5]. Approximately 0.4 mL of a heavy suspension of the bacterium *Escherichia coli* was added to each culture plate as a food source. The plates were incubated at a temperature of 23 C, with a 12 h light and dark cycle. Each plate was examined at least once a day for two weeks after the appearance of initial aggregations. Each isolate was purified and cultivated for taxonomic studies and preservation on non-nutrient water agar plates with *E. coli* pregrown for 12–24 h. Spores from these plates were frozen in HL 5 media [14] and stored at − 80 C in HMJAU.

### Morphological features and life cycle observations

Dictyostelid isolates were identified with the use of the descriptions provided by Raper [5], whose nomenclature also was followed except for those species recently assigned to new genera in the system of classification proposed by Sheikh et al. [11]. In the primary isolation plates, the location of each early aggregating clone and developing sorocarp was marked. The characteristic stages in the life cycle, including cell aggregation and the formation of pseudoplasmodia and ultimately sorocarps, were observed under a Zeiss dissecting microscope (Axio Zoom V16) with a 1.5× objective and a 10× ocular. Slides of sorocarps were prepared with water as the mounting medium. Characteristics of spores, sorophores, and sorocarps were observed and measured on the slides by using a Zeiss light microscope (Axio Imager A2), with a 10× ocular and 10, 40, and 100× (oil) objectives. Photographs were taken with a Zeiss Axiocam 506 color microscope camera.

Observation of spore germination. Hanging drop cultures, as described by Keller and Schoknecht [15], were prepared for observation of spore germination. Spores obtained from a sorus were mixed with a droplet of sterile water on the undersurface of a 22-mm square cover glass. The cover glass was then inverted over a depression slide. Vaseline was used to ring the edges of the cover glass. Spores were freely suspended in the water droplet. Characteristics of the myxamoebae were observed and photographed with a Zeiss light microscope (Axio Imager A2), using the 10× ocular and 10, 40, and 100× (oil) objectives.

### Nomenclature

According to the International Code of Nomenclature used for algae, fungi, and plants, the electronic version of this article in Portable Document Format (PDF) will represent a published work. In addition, new names contained in this study have been submitted to MycoBank and each will be allocated a unique MycoBank number which will be accessible through MycoBank, Index Fungorum, GBIF and other international biodiversity initiatives, where they will be made available to the Global Names Index.

### DNA isolation, PCR amplification and sequencing

After amoebae had cleared *E. coli* on the water agar media, the spores of the particular dictyostelid isolate being studied were collected with a sterile tip, mixed with the lysed buffer of the MiniBEST Universal Genomic DNA Extraction Kit Ver.5.0 (Takara, Japan) following the manufacturer’s protocol. The genomic DNA was used directly for the 18S PCR amplification using the primers 18SF-A (AACCTGGTTGATCCTGCCAG) and 18SR-B (TGATCCTTCTGCAGGTTCAC) [16] along with D542F (ACAATTGGAGGGCAAGTCTG3) and D1340R (TCGAGGTCTCGTCCGTTATC) [9]. PCR products were sent to Sangon Biotech Co., Ltd. (Shanghai, China) for sequencing. Sequences obtained were deposited at GenBank database. The isolates and the NCBI GenBank accession numbers of SSU DNA sequences used in present study are listed in Table 1.

**Table 1:** Species of dictyostelids included in the present study. Information is provided on the samples considered and the GenBank accession number for SSU sequences

Species	Voucher specimen	GenBank accession number (SSU)
*Acytostelium amazonicum* Cavender & Vadell	HN1B1	HQ141511.1
*A. leptosomum* Raper	FG12	AM168111.1
*A. serpentarium* Cavender et al.	SAB3A	AM168113.1
*A. subglobosum* Cavender	LB1	AM168110.1
*Cavenderia delicata* (H. Hagiw.) S. Baldauf, S. Sheikh & Thulin	TNS-C-226	AM168093.1
*C. fasciculata* (F. Traub et al.) S. Baldauf, S. Sheikh & Thulin	SH3	AM168087.1
*C. myxobasis* (Cavender et al.) S. Baldauf, S. Sheikh & Thulin	NT2A	HQ141522.1
*Coremiostelium polycephalum* (Raper) S. Baldauf, S. Sheikh, Thulin & Spiegel	MY1–1	AM168056.1
*C. polycephalum*	Landolt #1130 SS3B	HQ141488.1
*Dictyostelium brunneum* Kawabe	WS700	AM168031.1
*D. crassicaule* H.Hagiw.	93HO-33	AM168037.1
*D. purpureum* Olive	C143	AM168060.1
*D. purpureum* var. *pseudosessile* Li Y, P Liu, Y Zou SL Stephenson et JG Hou	4446	MH280023^a^
*D. purpureum* var. *pseudosessile*	4637	MH280022^a^
*D. pseudobrefeldianum* H.Hagiw.	91HO-8	AM168059.1
*D. robustum* H.Hagiw.	TNS-C-219	AM168064.1
*D. rosarium* Raper & Cavender	M45	AM168065.1
*D. septentrionale* Cavender	AK2	AM168067.1
*Hagiwaraea coeruleostipes* (Raper & Fennell) S. Baldauf, S. Sheikh & Thulin	CRLC53B	AM168036.1
*H. radiculata* (Cavender et al.) S. Baldauf, S. Sheikh & Thulin	ML5A	HQ141494.1
*H. vinaceofusca* (Raper & Fennell) S. Baldauf, S. Sheikh & Thulin	CC4	AM168062.1
*Heterostelium anisocaule* (Cavender et al.) S. Baldauf, S. Sheikh & Thulin	NZ47B	AM168096.1
*H. colligatum* (Vadell & Cavender) S. Baldauf, S. Sheikh & Thulin	HN13C1	HQ141505.1
*H. filamentosum* (F.Traub et al.) S. Baldauf, S.Sheikh & Thulin	SU-1	AM168100.1
*H. oculare* (Cavender et al.) S. Baldauf, S. Sheikh & Thulin	–	HQ141497.1
*Polysphondylium laterosorum* (Cavender) S. Baldauf, S. Sheikh & Thulin	AE4	AM168046.1
*P. violaceum* Bref.	209	HQ141486.1
*Raperostelium gracile* (H.Hagiw.) S. Baldauf, S. Sheikh & Thulin	TNS-C-183	AM168078.1
*R. monochasioides* (H.Hagiw.) S. Baldauf, S. Sheikh & Thulin	HAG653	AM168052.1
*R. potamoides* (Cavender et al.) S. Baldauf, S. Sheikh & Thulin	FP1A	AM168069.1
*R. tenue* (Cavender et al.) S. Baldauf, S. Sheikh & Thulin	PJ6	AM168094.1
*Rostrostelium ellipticum* (Cavender) S. Baldauf, S. Sheikh & Thulin	AE2	AM168112.1
*Synstelium polycarpum* (F.Traub et al.) S. Baldauf, S. Sheikh & Thulin	VE1b	AM168057.1
*S. polycarpum*	OhioWILDS	AM168058.1
*Tieghemostelium lacteum* (Tiegh.) S. Baldauf, S. Sheikh & Thulin	–	AM168045.1
*T. menorah* (Vadell & Cavender) S. Baldauf, S. Sheikh & Thulin	M1	AM168073.1

^a^samples and sequences obtained in the present study

### Phylogenetic analysis

The newly-generated sequences were checked and then submitted to GenBank. The ITS and SSU sequences were aligned and compared separately using the program MUSCLE v.3.6 [17, 18] and then manually adjusted in MEGA 7.0 [19]. Maximum likelihood (ML) analyses were performed using RAxML v7 [20]. In the ML analyses, the best-fit substitution models were estimated using GTR submission model and a gamma correction for rate variation among sites (GTRGAMMA), using the CIPRES server. The statistical support of clades was assessed with 1000 rapid-bootstrap (BS) replications.

## Additional file

Additional file 1: **Fig. S1.** Mixed culture of *Dictyostelium purpureum* var. *pseudosessile* with *D. purpureum*. A–C Aggregations. D Pseudoplasmodia. F Sorocarps. Scale bars: A,B,D: 1 mm, C: 500 μm, E: 2 mm. **Fig. S2.** Blast of SSU sequences of *Dictyostelium purpureum* var. *pseudosessile* with *D. purpureum* which have 97% identity. **Fig. S3.** Life cycle of *Dictyostelium purpureum*. The time of each stage showed on the top right corner. A–D Aggregations. E,F Pseudoplasmodia. G–L Sorocarps. Scale bars: A–H: 2 mm, I–L: 1 mm. (DOCX 344 kb)
