# Supplementary material for: Dictyostelium purpureum var. pseudosessile, a new variant of dictyostelid from tropical China
Source: BMC Evol Biol. 2019 Mar 14;19:78. doi: 10.1186/s12862-019-1407-2 (PMC6419474; doi:10.1186/s12862-019-1407-2)
Supplement: Supplementary file 1 — Fig. S1. Mixed culture of Dictyostelium purpureum var. pseudosessile with D. purpureum. A–C Aggregations. D Pseudoplasmodia. F Sorocarps. Scale bars: A,B,D: 1 mm, C: 500 μm, E: 2 mm. Fig. S2. Blast of SSU sequences of Dictyostelium purpureum var. pseudosessile with D. purpureum which have 97% identity. Fig. S3. Life cycle of Dictyostelium purpureum. The time of each stage showed on the top right corner. A–D Aggregations. E,F Pseudoplasmodia. G–L Sorocarps. Scale bars: A–H: 2 mm, I–L: 1 mm. (DOCX 344 kb) [file 12862_2019_1407_MOESM1_ESM.docx]

***Dictyostelium purpureum* var. *pseudosessile,* a new variant of dictyostelid from tropical China**

Pu Liu^1+^, Yue Zou^1+^, Jiangan Hou^1^, Steven L. Stephenson^2^, Yu Li^1^

^1^ *Engineering Research Center of Chinese Ministry of Education for Edible and Medicinal Fungi, Jilin Agricultural University, Changchun, 130118, P. R. China*

^2^ *Department of Biological Sciences, University of Arkansas, Fayetteville, AR 72701, USA*


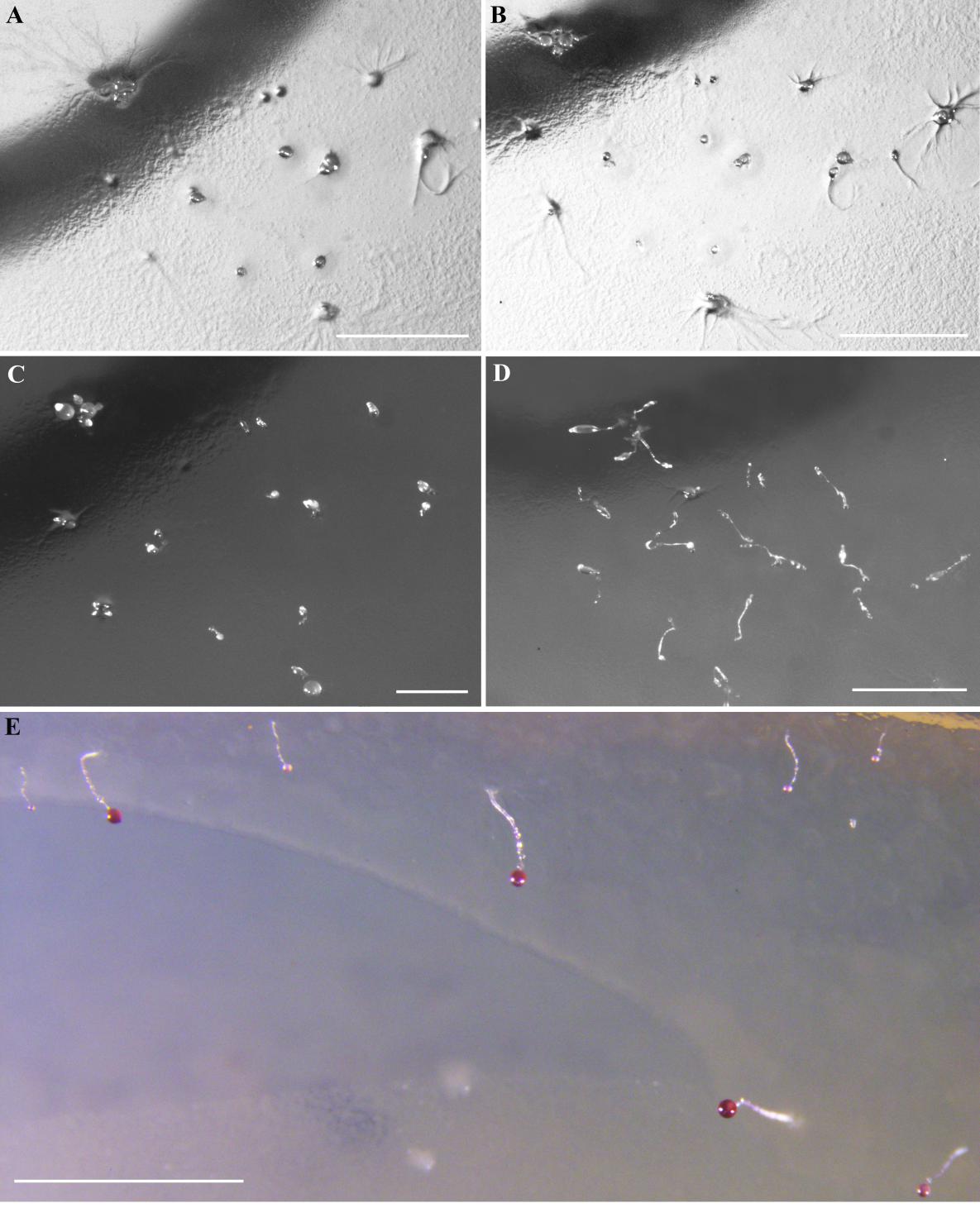


**Additional file 1: Figure S1** Mixed culture of *Dictyostelium purpureum* var. *pseudosessile* with *D. purpureum*. A–C Aggregations. D Pseudoplasmodia. F Sorocarps. Scale bars: A,B,D: 1 mm, C: 500 µm, E: 2 mm.


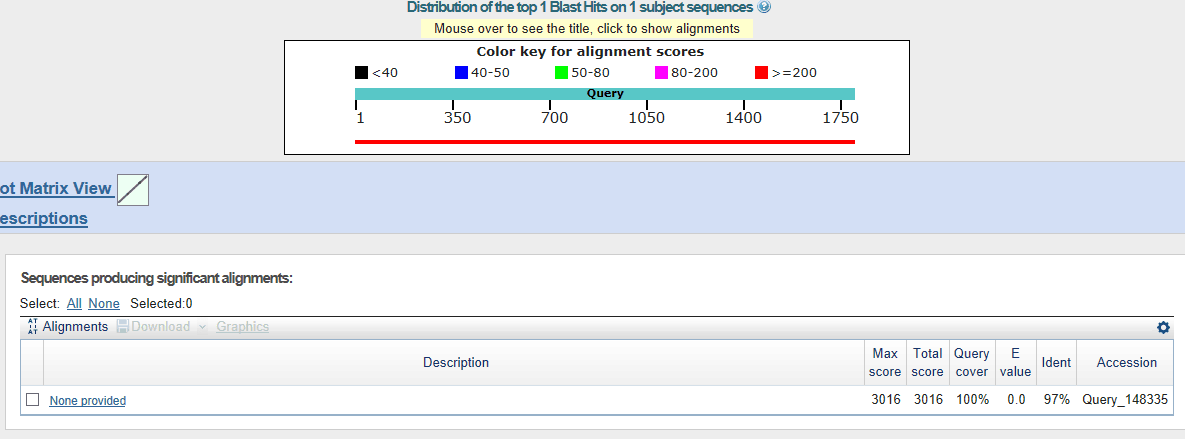

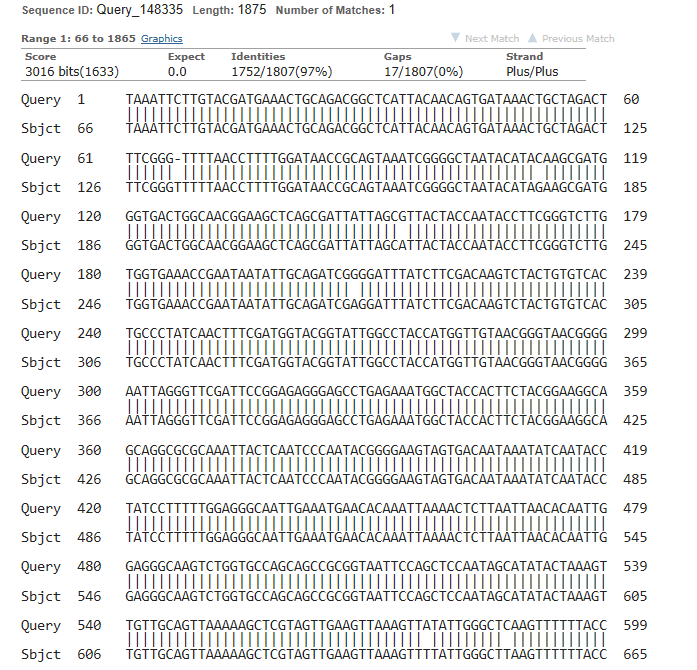

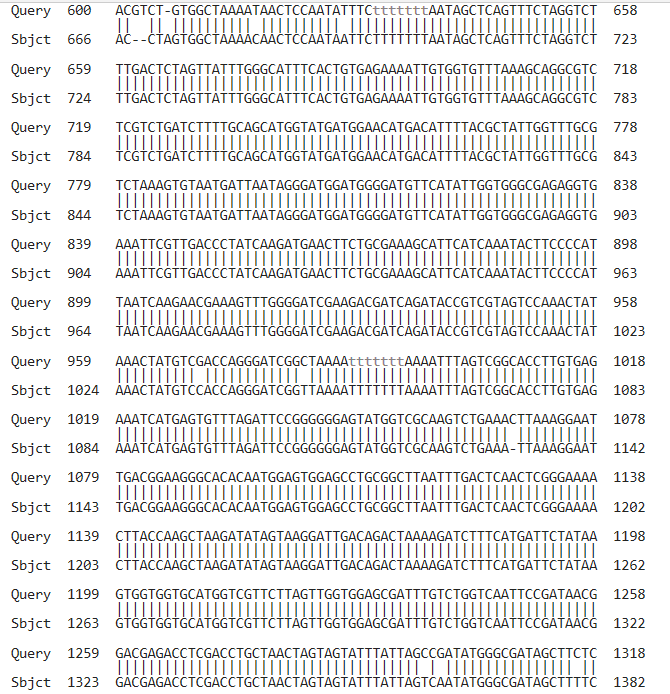

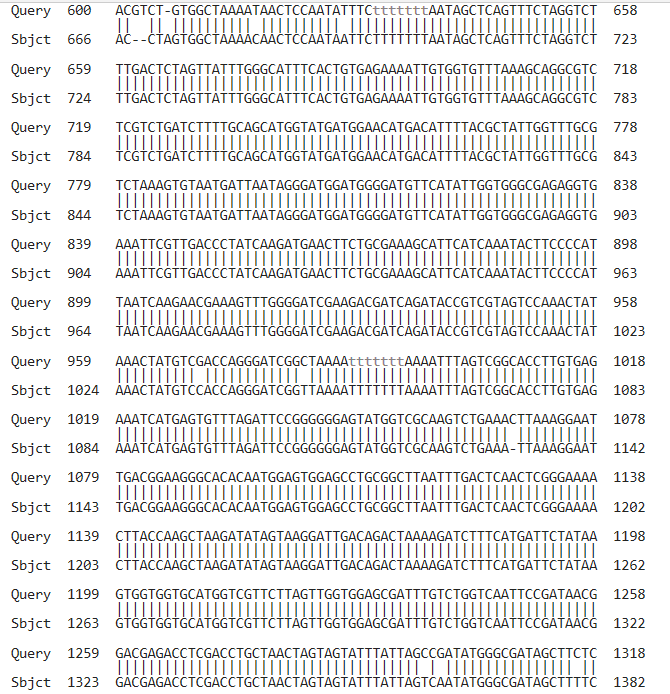

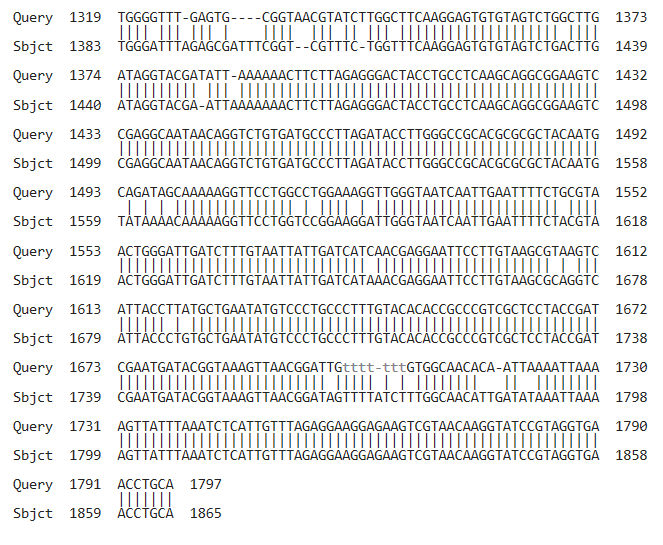


**Additional file 1: Figure S2** Blast of SSU sequences of *Dictyostelium purpureum* var. *pseudosessile* with *D. purpureum* which have 97 % identity.

**
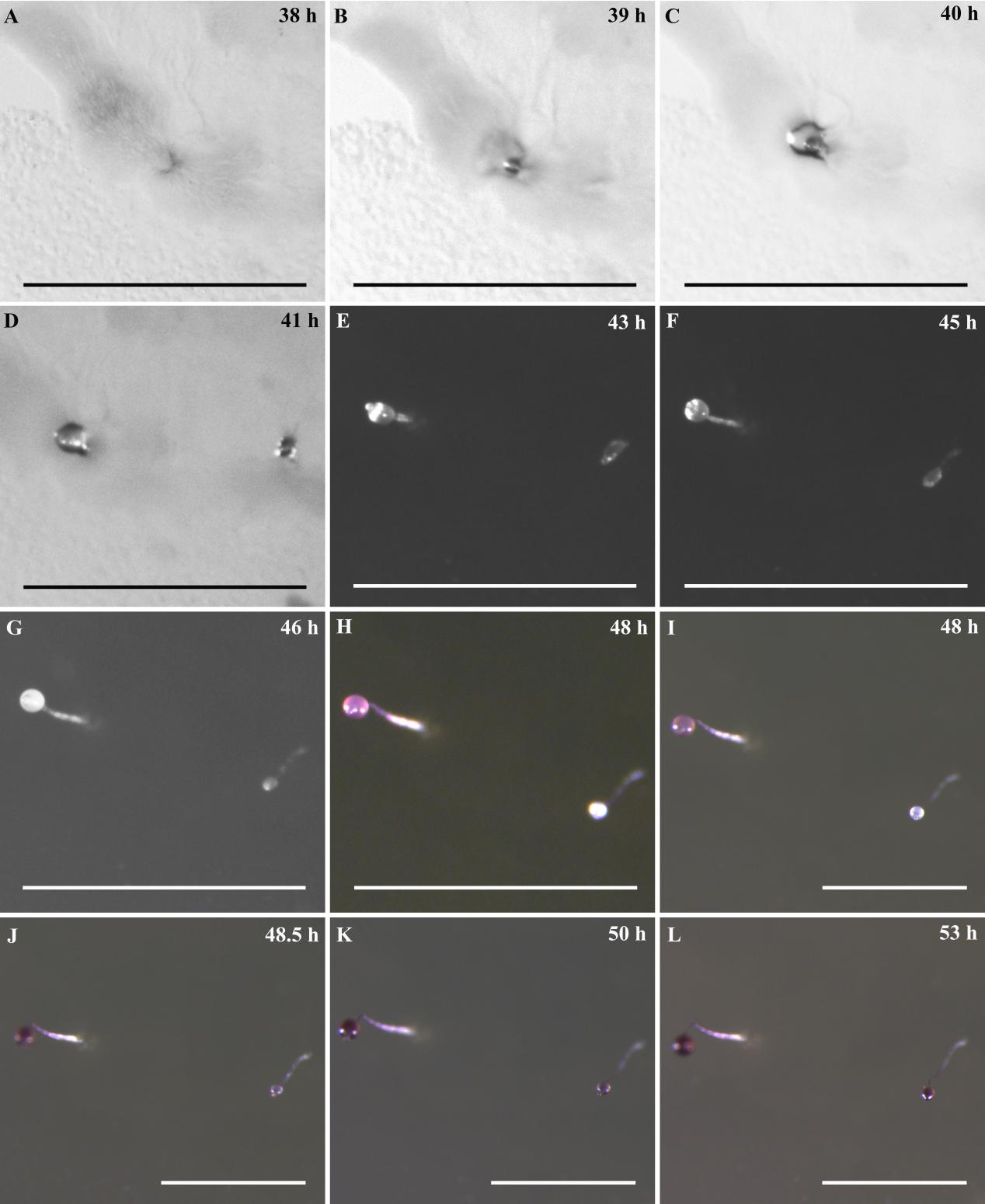
**

**Additional file 1: Figure S3** Life cycle of *Dictyostelium* *purpureum*. The time of each stage showed on the top right corner. A–D Aggregations. E,F Pseudoplasmodia. G–L Sorocarps. Scale bars: A–H: 2mm, I–L: 1 mm.
